# Association between estimated glucose disposal rate and history of cataract surgery: A population-based cross-sectional study from NHANES

**DOI:** 10.1097/MD.0000000000051292

**Published:** 2026-10-30

**Authors:** Guoan Liu, Yanman Zhong, Ruimei Qin, Jiaying Wang, Jinling Xie

**Affiliations:** a Department of Ophthalmology, Xinhui District People’s Hospital, Jiangmen, China; b Department of Clinical Laboratory, Xinhui District People’s Hospital, Jiangmen, China.

**Keywords:** cataract surgery rate, estimated glucose disposal rate (eGDR), insulin resistance, XGBoost-SHAP

## Abstract

This study aimed to investigate the association between estimated glucose disposal rate (eGDR) and cataract surgery, and to evaluate the predictive performance of eGDR using machine learning approaches. Using a retrospective cross-sectional design, the present study analyzed data sourced from the 2001–2008 National Health and Nutrition Examination Survey, which encompassed a total of 41,658 participants. Propensity score matching was used to balance the following covariates: age, gender, race, and the poverty-to-income ratio. The analytical methods used in this study included logistic regression and restricted cubic splines to examine nonlinearity relationships. Additionally, eXtreme Gradient Boosting-SHapley Additive exPlanations (SHAP) modeling was used to evaluate the importance of the predictors. An inverse dose-dependent relationship was observed: for each unit increase in eGDR, the odds of undergoing cataract surgery decreased (odds ratio = 0.914, 95% confidence interval: 0.875–0.954, *P* < .001). The highest eGDR quartile showed the strongest protective effect (Q4 vs Q1: odds ratio = 0.69, *P* = .0087). Restricted cubic spline analysis revealed a threshold of eGDR = 5.693 mg/kg/minutes. Above this threshold, the cataract surgery rate decreased linearly. Stratified analyses revealed a significant protective effect in individuals aged ≥60 years (*P* = .001), and observed stronger inverse associations in high-income subgroups (*P* = .02). eXtreme Gradient Boosting-SHAP modeling identified eGDR as a pivotal predictor (mean SHAP = 0.008), surpassing the performance of the traditional clinical indices. Elevated eGDR levels are associated with a lower rate of cataract surgery, which is modulated by age and socioeconomic status.

## 1. Introduction

Cataracts refer to the opacification of the lens that obstructs the normal transmission and focusing of light onto the retina, resulting in impaired vision.^[1]^ Metabolic dysregulation, abnormalities in protein synthesis and translation, as well as environmental and nutritional factors, are primary causes leading to microstructural changes in the lens and reduced light-transmitting capacity.^[2]^ Cataracts are a leading cause of blindness worldwide. They account for 47.8% to 51% of all blindness cases, as well as nearly 80% of avoidable blindness. An estimated 95 million people are affected by cataracts globally, with 35.5% of female patients and 30.1% of male patients experiencing blindness due to this condition.^[3,4]^ Despite increasing global cataract surgical rates in recent years, approximately 20 million people remain bilaterally blind from cataracts, predominantly concentrated in developing countries.^[5]^ It is noteworthy that cataract surgery has been consistently demonstrated to induce or exacerbate dry eye syndrome, regardless of the specific surgical technique used, including phacoemulsification, manual small-incision cataract surgery, or extracapsular cataract extraction.^[6,7]^ Consequently, early prevention, risk factor assessment, and management of cataracts are critical for reducing their associated socioeconomic and health burdens.

Insulin resistance (IR) is a critical factor in various metabolic disorders and has been confirmed to be related to an increased risk of cataract formation.^[8,9]^ One study found that age, high body mass index (BMI), and hypertension are the main risk factors for cataracts.^[10]^ The estimated glucose disposal rate (eGDR) is a validated composite index integrating blood pressure, waist circumference (WC), and glycated hemoglobin A1c (HbA1c). The eGDR is an innovative, cost-effective, and noninvasive index for quantifying IR,^[11,12]^ and offers greater clinical practicality than the normoglycemic hyperinsulinemic clamp technique for large-scale studies.^[13]^ Reduced eGDR values are associated with an increased risk of systemic diseases (including acute coronary syndrome, heart failure, fatty liver disease, and stroke) and adverse clinical outcomes.^[14–19]^ Additionally, hypertension, diabetes, and elevated HbA1c represent key metabolic factors implicated in cataract pathogenesis.^[20,21]^ The eGDR serves as a validated quantitative index of IR, representing a core feature preceding diabetes development. This establishes a foundation for linking eGDR to cataract progression. Investigating this relationship could lead to more effective preventive strategies and reduce the surgical burden associated with cataracts.

The present study investigated the association between eGDR and the history of cataract surgery using cross - sectional analyses of National Health and Nutrition Examination Survey (NHANES) data from 2001 to 2008. Through multivariable-adjusted analyses that incorporated propensity score matching (PSM), logistic regression analysis, and an eXtreme Gradient Boosting (XGBoost) algorithm, we quantitatively assessed the epidemiological association of this metabolic parameter. Comprehensive sensitivity analyses confirmed the robustness of our findings. These findings provide insight into the potential role of eGDR in the history of cataract surgery and could inform precision prevention strategies in public health.

## 2. Materials and methods

### 2.1. Data source and preprocessing procedures

The NHANES is an ongoing, cross-sectional, nationally representative survey conducted in the United States. As a key initiative of the National Center for Health Statistics, NHANES is funded and supported by the Centers for Disease Control and Prevention to evaluate the health and nutritional condition of non-institutionalized U.S. residents. The survey collects data biennially using a complex, multistage probability sampling design. This includes in-home interviews followed by physical examinations at Mobile Examination Centers, where blood and urine specimens are collected. NHANES protocols were reviewed and approved by the National Center for Health Statistics Research Ethics Review Board, with written informed consent obtained from all participants. For this study, we used NHANES data from 2001–2008 cycles containing comprehensive history of cataract surgery data.

From the initial cohort of 41,658 participants, exclusion criteria were applied as follows: exclusion of participants aged <20 years (n = 20,292); removal of cases with incomplete history of cataract surgery data (n = 5385); and elimination of subjects with missing or invalid eGDR measurements or extreme values beyond biologically plausible ranges (n = 2823). The final analytical cohort included 13,158 eligible individuals. Of those, 1540 had a history of cataract surgery, while the remaining 11,618 served as controls. The complete selection workflow is detailed in Figure 1.

**Figure 1.**
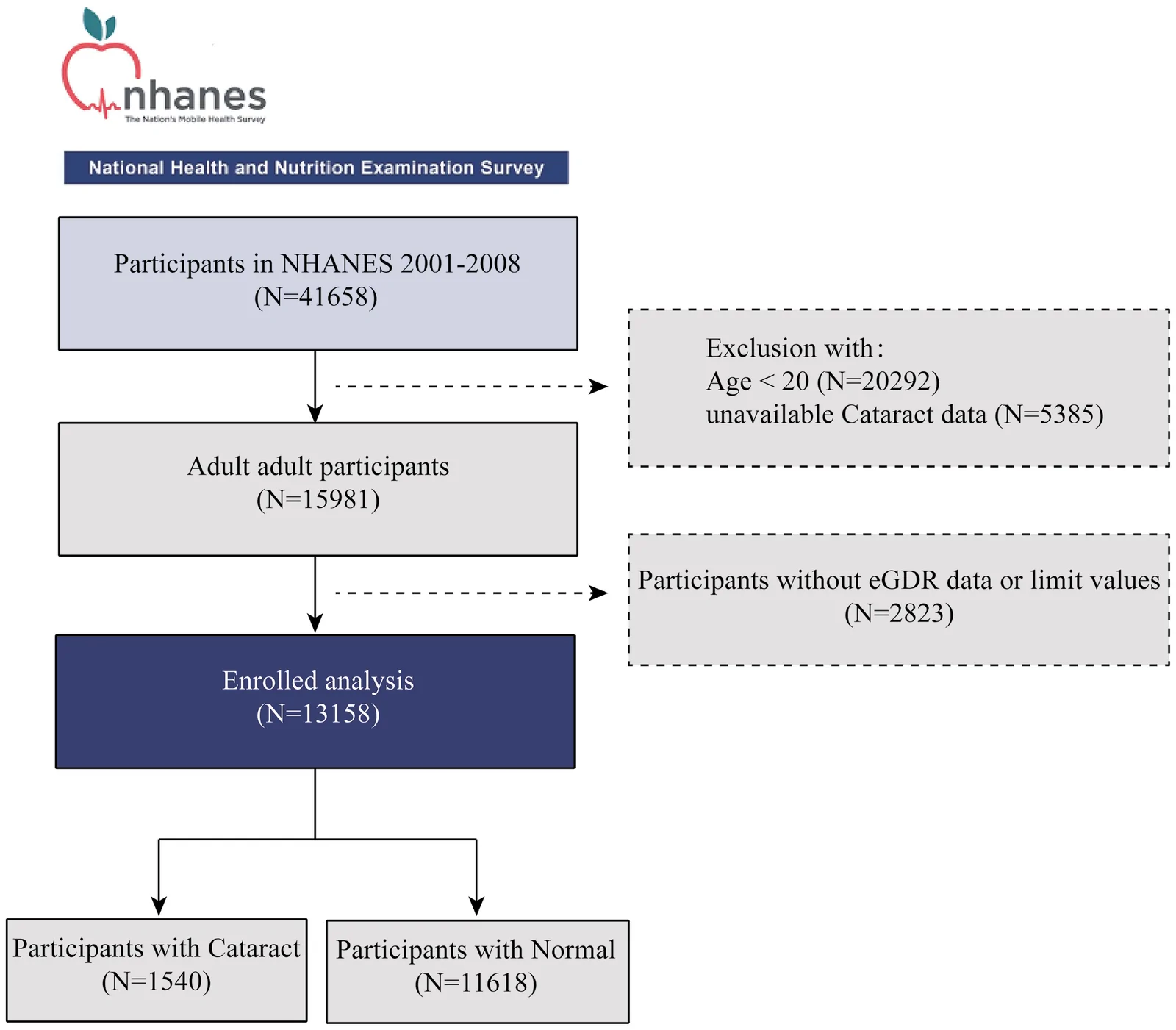
Flowchart of the sample selection from the NHANES 2001–2008. NHANES = National Health and Nutrition Examination Survey.

### 2.2. Case identification of history of cataract surgery

In alignment with the conventions of epidemiological studies, we used a self-reported history of cataract surgery to identify cataract-positive cases in the present study. The study population was restricted to community-dwelling adults aged 20 years and older. History of cataract surgery was operationalized as the dependent variable in the analytical models. The method of case identification is consistent with that documented in previous studies.^[22–24]^ This method aligns with Centers for Disease Control and Prevention surveillance protocols for epidemiological studies of the eye.

### 2.3. Estimated glucose disposal rate (eGDR)

The eGDR was originally established as a validated metric for assessing IR in type 1 diabetes.^[11]^ This composite parameter integrates hypertension status, WC, and HbA1c values. The eGDR is calculated using the following formula^[12]^: eGDR (mg/kg/minutes) = 21.158–(0.090 × WC)–(3.407 × hypertension status)–(0.551 × HbA1c). In this formula: WC is measured in centimeters, hypertension status is coded as present (assigned 1) or absent (assigned 0); and HbA1c is expressed as a percentage. These parameters were chosen because of their established associations with insulin sensitivity. Compared to the hyperinsulinemic-euglycemic clamp technique, this methodology retains diagnostic reliability while offering significantly enhanced practicality for large-scale clinical studies.^[13]^

### 2.4. Covariate selection

Covariate selection was based on statistically significant associations from previous literature, incorporating demographic factors (e.g., age strata, gender, race/ethnicity, family poverty-to-income ratio [PIR]), metabolic parameters (e.g., BMI, HbA1c, total cholesterol, renal biomarkers like urea nitrogen, urinary albumin, creatinine), and hematologic indices (e.g., leukocyte/erythrocyte counts, mean corpuscular hemoglobin concentration (MCHC), potassium). The analyses were exploratory in nature, and the adjusted estimates are intended to describe associations rather than to support definitive causal conclusions.

Variables with a missing rate exceeding 50% (e.g., systolic blood pressure, 72.3%; fasting glucose, 51.1%; insulin, 97.9%; and fibrinogen, 84.8%) were excluded from the analytical models to avoid producing unstable estimates. All variables were rigorously operationalized within multivariable-adjusted models that strictly adhered to NHANES protocols to effectively mitigate confounding factors. Key demographic variables, including age, gender, race/ethnicity (grouped into: Mexican American, Non-Hispanic Black, Non-Hispanic White, Other Hispanic, and Other/Multiracial), and family PIR, were sourced directly from the NHANES demographic files. Socioeconomic status was stratified into low (PIR ≤ 1.3), middle (PIR 1.3–3.5), and high (PIR > 3.5) income groups based on federal poverty thresholds. Diabetes and hypertension were assessed via self-reported physician diagnoses from questionnaires. Laboratory parameters, such as leukocyte count, urea nitrogen, urinary albumin, total cholesterol, HbA1c, erythrocyte count, MCHC, potassium, and creatinine, were derived from NHANES laboratory assays.

### 2.5. Propensity score matching (PSM)

To ensure comparable baseline characteristics between cataract surgery cases and controls while minimizing confounding effects, 1:1 PSM was implemented using logistic regression models that incorporated age strata, biological gender, race/ethnicity, and family PIR. Post-matching covariate balance was verified through standardized mean differences, with an optimal threshold set below 0.1. Subsequent comparative analyses were performed on the matched cohorts to validate the robustness of the ophthalmological outcome measures. This methodological approach effectively addressed selection bias inherent in observational cataract studies while preserving sample representativeness, in accordance with STROBE guidelines for non-randomized research.

### 2.6. Logistic regression analysis

The association between eGDR and history of cataract surgery was evaluated using binary logistic regression analysis. Analyses treated eGDR both as a continuous variable (per unit increment) and as a categorical variable stratified by interquartile ranges (Q1–Q4). Three hierarchical models were constructed: a crude, unadjusted model; Model 1, which was adjusted for demographic covariates (age strata, gender, race/ethnicity, and family PIR); and Model 2, which additionally incorporated metabolic parameters (BMI, HbA1c, and total cholesterol), renal parameters (urea nitrogen, urinary albumin, and creatinine), and hematologic parameters (leukocyte and erythrocyte counts, MCHC, and potassium). Because this study aimed to estimate exposure-outcome associations rather than national prevalence, and used PSM to control confounding, the matched sample no longer represents the national population; complex survey weights were therefore not applied in the analytical models. Continuous variables that did not follow normal distributions were transformed using weighted quantile-based categorization. Categorical analyses yielded odds ratios (ORs) alongside corresponding 95% confidence intervals (CIs). The first quartile (Q1) was used as the reference category. Effect modification was assessed via stratified interaction testing, with adjustments for multiple comparisons conducted using the Benjamini-Hochberg procedure to control the false discovery rate. This approach enhances the validity of our inference in this nationally representative sample.

### 2.7. XGBoost analysis

The research used XGBoost, an optimized version of gradient-boosted decision trees that sequentially combines multiple weak, tree-based models to improve predictive performance. This machine learning approach was used to evaluate the contribution weights of various factors associated with cataract surgery. To optimize the model, key parameters were carefully tuned. The boosting step size was set to 0.08 to balance learning capacity and generalization ability. Additionally, overfitting control measures were implemented through regularization techniques, such as limiting tree complexity (maximum depth = 6) and using instance sampling (80% per iteration). These measures ensured reliable and consistent analytical results.

### 2.8. Clinical subgroup analysis

Subgroup analyses assessed potential effect modification in the eGDR-cataract surgery association across clinically relevant strata, including categorical age groups, gender, race/ethnicity, family PIR, smoking status, diabetes, and cardiovascular disease (CVD). Interaction testing evaluated heterogeneity in exposure-outcome effect sizes across stratified subgroups. The results were visualized using forest plots to compare ORs and 95% CIs, offering a clear illustration of variations in effect size and the consistency of associations.

### 2.9. Sensitivity analysis

To address missing data, multiple imputation was used in the sensitivity analyses of this study. To validate the stability of the association between eGDR and cataract surgery, individuals with diabetes, a well-known risk factor for cataracts, were excluded. Chronic hyperglycemia can lead to lens degeneration and damage, thereby increasing the risk of cataracts. A comparative analysis revealed that the inverse relationship between eGDR and cataract surgery persisted consistently, even after excluding diabetic participants, thereby further supporting the reliability of the initial findings.

### 2.10. Statistical analysis

All data processing and analytical procedures were conducted using the R software (version 4.1.1). For non-normally distributed continuous variables, data were presented as medians accompanied by their interquartile ranges (IQRs), while categorical variables were expressed as counts alongside their corresponding relative frequencies. Differences across eGDR quartiles were evaluated using Wilcoxon rank-sum tests for continuous variables and chi-square tests for categorical variables. Multiple imputation with five imputation matrices was used to address the missing values, which were less than 20%. Convergence of the imputation procedure was assessed by visual inspection of trace plots for key variables (Fig. S1, Supplemental Digital Content 1), which confirmed the stability of the imputed values across iterations. The missing-at-random assumption was further evaluated by comparing a complete-case sensitivity analysis (OR = 0.952, 95% CI 0.913–0.993) against the imputed analysis (OR = 0.932, 95% CI 0.898–0.967), which yielded consistent effect directions. The XGBoost algorithm was used to assess the relative importance of variables in predicting the cataract surgery and to identify critical predictive factors via gain-based feature importance ranking. All statistical tests were two-tailed. *P*-values <.05 were considered significant.

## 3. Results

### 3.1. Baseline characteristics of the samples

Missing values were handled using multiple imputation, with pre- and post- imputation consistency verified (Table S1, Supplemental Digital Content 2). The study initially enrolled 13,158 participants (1540 cataract surgery cases and 11,618 controls), but this number was reduced to 3080 following PSM (standardized differences <10% for demographics; shown in Fig. 2). Key changes from pre- to post-PSM included: The proportion of older adults increased from 44.56% to 95%; the gender balance was maintained (51.28% to 54% female); the percentage of Non-Hispanic Whites rose from 52.61% to 72%; and smoking status showed marginal significance *(P* = .083). Significant differences persisted in diabetes prevalence (non-diabetic: 85.42%–78%); hypertension prevalence (non-hypertensive: 61.52%–45%); and CVD prevalence (5.62%–11%). Several indexes displayed substantial intergroup variations: BMI (*P* < .001–.001), hemoglobin (*P* < .001 in both phases), glycated hemoglobin (*P* < .001 in both phases), and eGDR (*P* < .001–.002). Complete data are presented in Table 1, Table S2, Supplemental Digital Content 6, and Table S3, Supplemental Digital Content 7.

**Table 1:** Baseline characteristics of cataract cases in the NHANES.

Variable	Before PSM				After PSM			
	Overall, N = 13,158*	Cataract, N = 1540*	Normal, N = 11,618*	*P*-value†	Overall, N = 3080*	Cataract, N = 1540*	Normal, N = 1540*	*P*-value†
Age group				<.001				>.99
Old	5863 (44.56%)	1464 (95.06%)	4399 (37.86%)		2928 (95%)	1464 (95%)	1464 (95%)	
Young	7295 (55.44%)	76 (4.94%)	7219 (62.14%)		152 (4.9%)	76 (4.9%)	76 (4.9%)	
Gender				.008				>.99
Female	6748 (51.28%)	839 (54.48%)	5909 (50.86%)		1678 (54%)	839 (54%)	839 (54%)	
Male	6410 (48.72%)	701 (45.52%)	5709 (49.14%)		1402 (46%)	701 (46%)	701 (46%)	
Race				<.001				>.99
Mexican American	2436 (18.51%)	160 (10.39%)	2276 (19.59%)		320 (10%)	160 (10%)	160 (10%)	
Non-Hispanic Black	2489 (18.92%)	168 (10.91%)	2321 (19.98%)		336 (11%)	168 (11%)	168 (11%)	
Non-Hispanic White	6923 (52.61%)	1105 (71.75%)	5818 (50.08%)		2210 (72%)	1105 (72%)	1105 (72%)	
Other Hispanic	815 (6.19%)	72 (4.68%)	743 (6.40%)		144 (4.7%)	72 (4.7%)	72 (4.7%)	
Other Race	495 (3.76%)	35 (2.27%)	460 (3.96%)		70 (2.3%)	35 (2.3%)	35 (2.3%)	
PIR				<.001				>.99
≤1.3	3621 (27.52%)	429 (27.86%)	3192 (27.47%)		858 (28%)	429 (28%)	429 (28%)	
1.3–3.5	5164 (39.25%)	760 (49.35%)	4404 (37.91%)		1520 (49%)	760 (49%)	760 (49%)	
>3.5	4373 (33.23%)	351 (22.79%)	4022 (34.62%)		702 (23%)	351 (23%)	351 (23%)	
Smoking history				<.001				.083
Non-smoked	6637 (50.44%)	700 (45.45%)	5937 (51.10%)		1448 (47%)	700 (45%)	748 (49%)	
Smoked	6521 (49.56%)	840 (54.55%)	5681 (48.90%)		1632 (53%)	840 (55%)	792 (51%)	
BMI	28.69 (6.24)	27.93 (5.53)	28.79 (6.32)	<.001	28.2 (5.6)	27.9 (5.5)	28.6 (5.6)	.001
Weight, kg	66.43 (4.14)	65.57 (4.07)	66.55 (4.13)	<.001	65.9 (4.1)	65.6 (4.1)	66.2 (4.1)	<.001
Waist, cm	99.15 (15.21)	100.21 (14.18)	99.01 (15.34)	<.001	101 (14)	100 (14)	101 (14)	.18
Diabetes				<.001				<.001
No	11,240 (85.42%)	1133 (73.57%)	10,107 (86.99%)		2412 (78%)	1133 (74%)	1279 (83%)	
Borderline	234 (1.78%)	45 (2.92%)	189 (1.63%)		83 (2.7%)	45 (2.9%)	38 (2.5%)	
Yes	1684 (12.80%)	362 (23.51%)	1322 (11.38%)		585 (19%)	362 (24%)	223 (14%)	
Hypertension				<.001				<.001
No	8095 (61.52%)	634 (41.17%)	7461 (64.22%)		1386 (45%)	634 (41%)	752 (49%)	
Yes	5063 (38.48%)	906 (58.83%)	4157 (35.78%)		1694 (55%)	906 (59%)	788 (51%)	
Cardiovascular diseases				<.001				.21
No	12,418 (94.38%)	1353 (87.86%)	11,065 (95.24%)		2728 (89%)	1353 (88%)	1375 (89%)	
Yes	740 (5.62%)	187 (12.14%)	553 (4.76%)		352 (11%)	187 (12%)	165 (11%)	
Albumin urine, µg/mL	58.16 (481.83)	101.42 (564.29)	52.43 (469.55)	<.001	71 (425)	101 (564)	40 (202)	<.001
Hemoglobin, g/dL	14.24 (1.54)	13.89 (1.51)	14.29 (1.53)	<.001	14.07 (1.49)	13.89 (1.51)	14.26 (1.44)	<.001
Red blood cell count, million cells/µL	4.67 (0.51)	4.49 (0.50)	4.70 (0.50)	<.001	4.55 (0.50)	4.49 (0.50)	4.62 (0.49)	<.001
Mean cell Hgb conc, g/dL	34.01 (0.95)	33.90 (0.86)	34.03 (0.96)	<.001	33.93 (0.87)	33.90 (0.86)	33.96 (0.88)	.010
Mean cell hemoglobin, pg	30.55 (2.25)	31.04 (2.27)	30.48 (2.24)	<.001	30.98 (2.16)	31.04 (2.27)	30.93 (2.04)	.081
White blood cell count, 1000 cells/µL	7.25 (2.57)	7.33 (3.55)	7.24 (2.42)	.93	7.22 (2.99)	7.33 (3.55)	7.11 (2.30)	.042
Mean platelet volume, fL	8.03 (0.89)	8.05 (0.94)	8.03 (0.88)	.72	8.05 (0.92)	8.05 (0.94)	8.06 (0.90)	.59
Hematocrit (%)	41.86 (4.38)	40.96 (4.43)	41.98 (4.36)	<.001	41.5 (4.4)	41.0 (4.4)	42.0 (4.2)	<.001
C-reactive protein, mg/dL	0.47 (0.90)	0.54 (1.18)	0.46 (0.86)	<.001	0.52 (1.10)	0.54 (1.18)	0.51 (1.01)	.39
Total cholesterol, mg/dL	201.04 (42.11)	198.90 (43.89)	201.32 (41.86)	.016	202 (44)	199 (44)	204 (44)	<.001
Blood urea nitrogen, mg/dL	13.76 (6.34)	17.98 (8.16)	13.20 (5.84)	<.001	17 (8)	18 (8)	16 (7)	<.001
Total protein, g/dL	7.18 (0.50)	7.13 (0.50)	7.18 (0.50)	<.001	7.14 (0.50)	7.13 (0.50)	7.15 (0.50)	.61
Total bilirubin, mg/dL	0.75 (0.35)	0.77 (0.28)	0.75 (0.36)	<.001	0.78 (0.36)	0.77 (0.28)	0.78 (0.43)	.82
Chloride, mmol/L	103.58 (2.98)	102.93 (3.42)	103.67 (2.91)	<.001	103 (3)	103 (3)	103 (3)	.17
Aspartate aminotransferase AST, U/L	25.66 (12.64)	25.23 (8.69)	25.71 (13.07)	.013	25 (11)	25 (9)	25 (14)	.11
Total calcium, mg/dL	9.46 (0.38)	9.49 (0.42)	9.45 (0.38)	<.001	9.49 (0.41)	9.49 (0.42)	9.48 (0.39)	.77
Creatinine refrigerated serum, mg/dL	0.93 (0.42)	1.08 (0.58)	0.91 (0.39)	<.001	1.03 (0.47)	1.08 (0.58)	0.98 (0.31)	<.001
Alanine aminotransferase, U/L	24.95 (17.78)	21.36 (10.71)	25.43 (18.47)	<.001	22 (18)	21 (11)	23 (23)	<.001
Cholesterol refrigerated serum, mg/dL	202.03 (42.40)	199.66 (44.59)	202.34 (42.10)	.008	202 (44)	200 (45)	205 (44)	<.001
Lactate dehydrogenase, U/L	132.18 (27.88)	140.34 (29.43)	131.10 (27.49)	<.001	139 (29)	140 (29)	138 (29)	.006
Albumin refrigerated serum, g/dL	4.18 (0.35)	4.13 (0.32)	4.19 (0.36)	<.001	4.15 (0.31)	4.13 (0.32)	4.17 (0.29)	<.001
Potassium, mmol/L	4 (0.36)	4.14 (0.42)	3.98 (0.35)	<.001	4.10 (0.41)	4.14 (0.42)	4.07 (0.39)	<.001
Sodium, mmol/L	139.09 (2.47)	139.28 (2.74)	139.06 (2.43)	<.001	139 (3)	139 (3)	139 (3)	.79
Triglycerides refrig serum, mg/dL	154.18 (106.11)	154.54 (92.10)	154.13 (107.84)	.003	156 (95)	155 (92)	158 (98)	.43
Glycohemoglobin	5.71 (1.06)	5.96 (1.03)	5.68 (1.07)	<.001	5.89 (1.00)	5.96 (1.03)	5.82 (0.97)	<.001
eGDR	7.77 (2.62)	6.85 (2.44)	7.90 (2.62)	<.001	6.99 (2.45)	6.85 (2.44)	7.14 (2.46)	.002

PIR ≤ 1.3: low PIR; 1.3 < PIR ≤ 3.5: middle PIR; PIR > 3.5: high PIR.

eGDR = estimated glucose disposal rate, NHANES = National Health and Nutrition Examination Survey, PIR = poverty-to-income ratio, PSM = propensity score matching, SD = standard deviation.

* Mean (SD) or frequency (%).

† Wilcoxon rank-sum test; Pearson chi-squared test.

**Figure 2.**
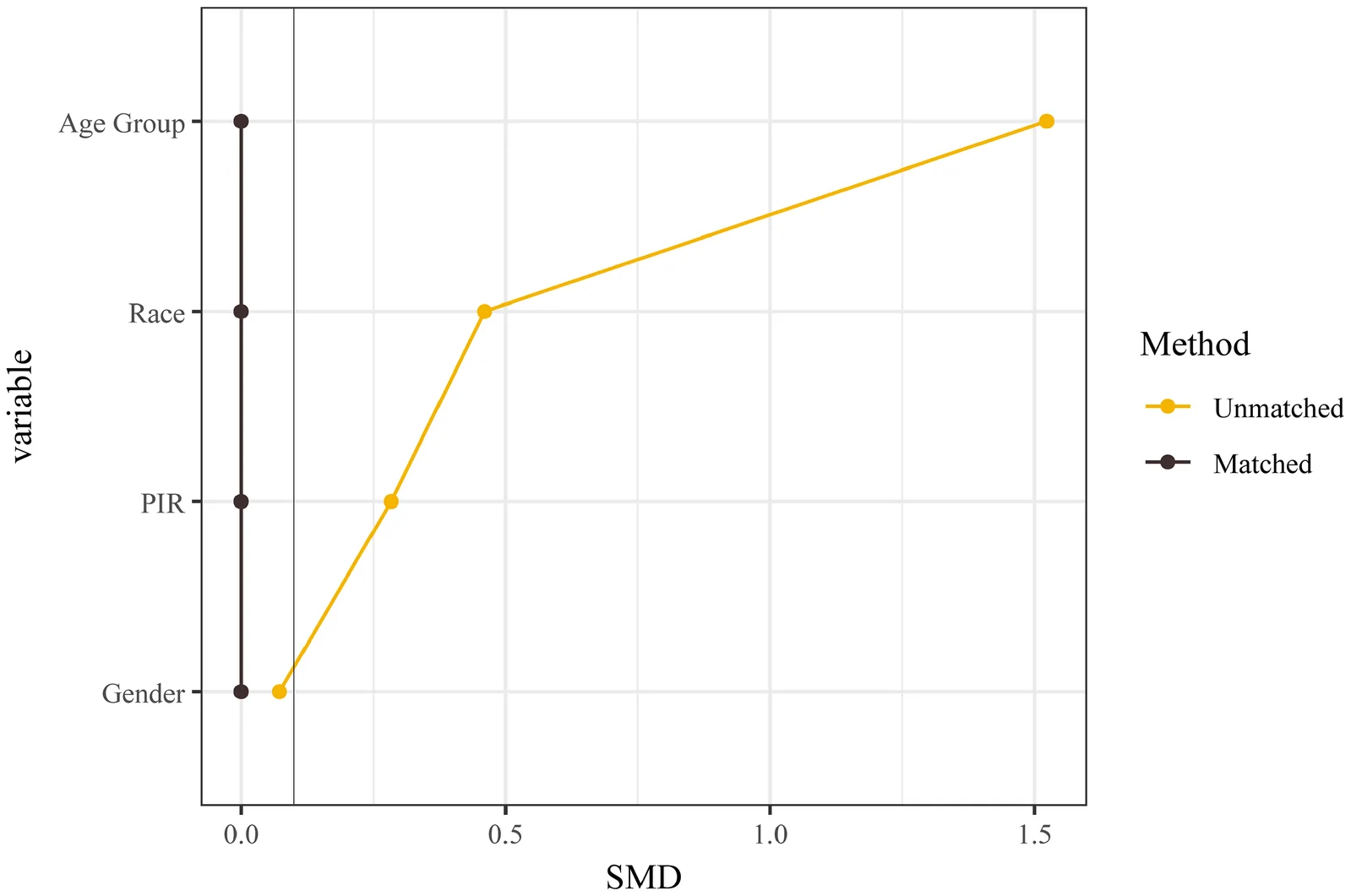
Evaluating covariate equilibrium pre- and post-PSM.

### 3.2. Association between eGDR and history of cataract surgery

Multivariable linear regression models were employed to analyze the association between eGDR and a history of cataract surgery. A significant negative correlation was found between eGDR and cataract surgery. In the crude model, each unit increase in eGDR (continuous) reduced cataract surgery (OR = 0.953, 95%CI: 0.926–0.981, *P* = .0011). This association remained significant after multi-factor adjustments: Model 1 (OR = 0.951, 95%CI: 0.923–0.980, *P* < .001) and Model 2 (OR = 0.914, 95%CI: 0.875–0.954, *P* < .001). The crude model further analyzed eGDR by quartiles, demonstrating that the highest quartile (Q4: eGDR ≥ 9.07) had a significantly lower rate of cataract surgery compared to the lowest quartile (Q1: eGDR < 5.16) (OR = 0.795, 95%CI: 0.651–0.971, *P* = .025). This protective trend was maintained in Model 1 and Model 2 for Q4 (OR = 0.787, 95%CI: 0.642–0.966, *P* = .0217 and OR = 0.690, 95%CI: 0.523–0.910, *P* = .0087, respectively). Significant trend tests were observed across the models (crude: *P* = .0062; Model 1: *P* = .0053; Model 2: *P* = .0013), suggesting that elevated eGDR levels offer protection against cataract surgery (Table 2).

**Table 2:** Univariate logistic regression analysis results.

Characterisitic	Exposure cutoff	Case (%)	OR	Crude Model			Model 1			Model 2	*P*-value
				95%CI	*P*-value	OR	95%CI	*P*-value	OR	95%CI	
eGDR											
eGDR continuous			0.953	(0.926,0.981)	.0011	0.951	(0.923,0.98)	*P* < .001	0.914	(0.875,0.954)	*P* < .001
eGDR quantile											
Q1(low)	< 5.16	770(25%)	Ref	Ref		Ref	Ref		Ref	Ref	
Q2	5.16-< 6.77	770(25%)	1.104	(0.904,1.349)	.3322	1.101	(0.901,1.347)	0.3476	1.013	(0.801,1.281)	.9125
Q3	6.77-< 9.07	770(25%)	0.906	(0.742,1.106)	.3329	0.901	(0.737,1.102)	0.311	0.84	(0.661,1.068)	.155
Q4(high)	≥ 9.07	770(25%)	0.795	(0.651,0.971)	.025	0.787	(0.642,0.966)	0.0217	0.69	(0.523,0.91)	.0087
p for trend					.0062			0.0053			.0013

eGDR: Q1: eGDR < 5.16; Q2: 5.16 ≤ eGDR < 6.77; Q3: 6.77 ≤ eGDR < 9.07; Q4: eGDR ≥ 9.07.

The Crude Model was non-adjusted.

The Model 1 was adjusted by Age Group, Gender, Race, PIR.

The Model 2 was adjusted by Age Group, Gender, Race, PIR, White blood cell count, BMI, Blood Urea Nitrogen, Albumin urine, Cholesterol refrigerated serum, Glycohemoglobin, Red blood cell count, Mean Cell Hgb Conc, Potassium, Creatinine refrigerated serum.

CI = confidence intervals, eGDR = Estimated Glucose Disposal Rate, OR = odds ratio.

### 3.3. Machine learning-based predictive analysis of cataract surgery related factors via XGBoost algorithm

To evaluate the relative contributions of various variables to cataract surgery, the XGBoost model was employed, and a thorough interpretation was offered via Shapley Additive Explanations (SHAP) analysis (Fig. 3). The model showed great predictive accuracy, with an AUC of 0.85. It had a mean squared error of 0.08, showing slight squared deviation between predicted and actual values. It also had a root mean squared error of 0.28, which further confirmed its accuracy. For comparison, a multivariable logistic regression model fitted on the same data yielded an mean squared error of 0.08 and an root mean squared error of 0.28 (AUC = 0.86), indicating that the two approaches performed comparably (Fig. S2, Supplemental Digital Content 3). Therefore, the added value of the XGBoost model lies primarily in its SHAP-based interpretability and variable importance ranking, rather than in superior predictive accuracy. The variable importance rankings indicated that eGDR is a significant predictor (with a mean SHAP value of 0.008), placing it just below red blood cell count, urinary albumin, and blood urea nitrogen, yet above other variables such as MCHC (0.0043), white blood cell count (0.0034), and total cholesterol (0.0032). Although the mean SHAP magnitude of eGDR (0.008) was numerically smaller than that of the red blood cell count and several other parameters, eGDR was designated as pivotal because it is a single, clinically modifiable composite index of IR that integrates glycemic, anthropometric, and hemodynamic dimensions, whereas red blood cell count and several other parameters represent a nonspecific hematologic marker of non-IR with limited direct relevance to insulin metabolic intervention. As a novel composite metric, eGDR showed a significant negative correlation in the model, highlighting its strong association with cataract surgery (Fig. 3A). The SHAP dependence plots further clarified the variable-specific contributions (Fig. 3B): eGDR showed a significant negative correlation, with decreasing SHAP values at higher eGDR levels corresponding to reduced cataract surgery; urinary albumin and glycated hemoglobin displayed positive correlations, whereas the red blood cell count showed a negative correlation. Socioeconomic (PIR: 0.001), gender (0.0007), and race (0.0005) factors exhibited lower, yet significant, predictive contributions based on SHAP value distributions.

**Figure 3.**
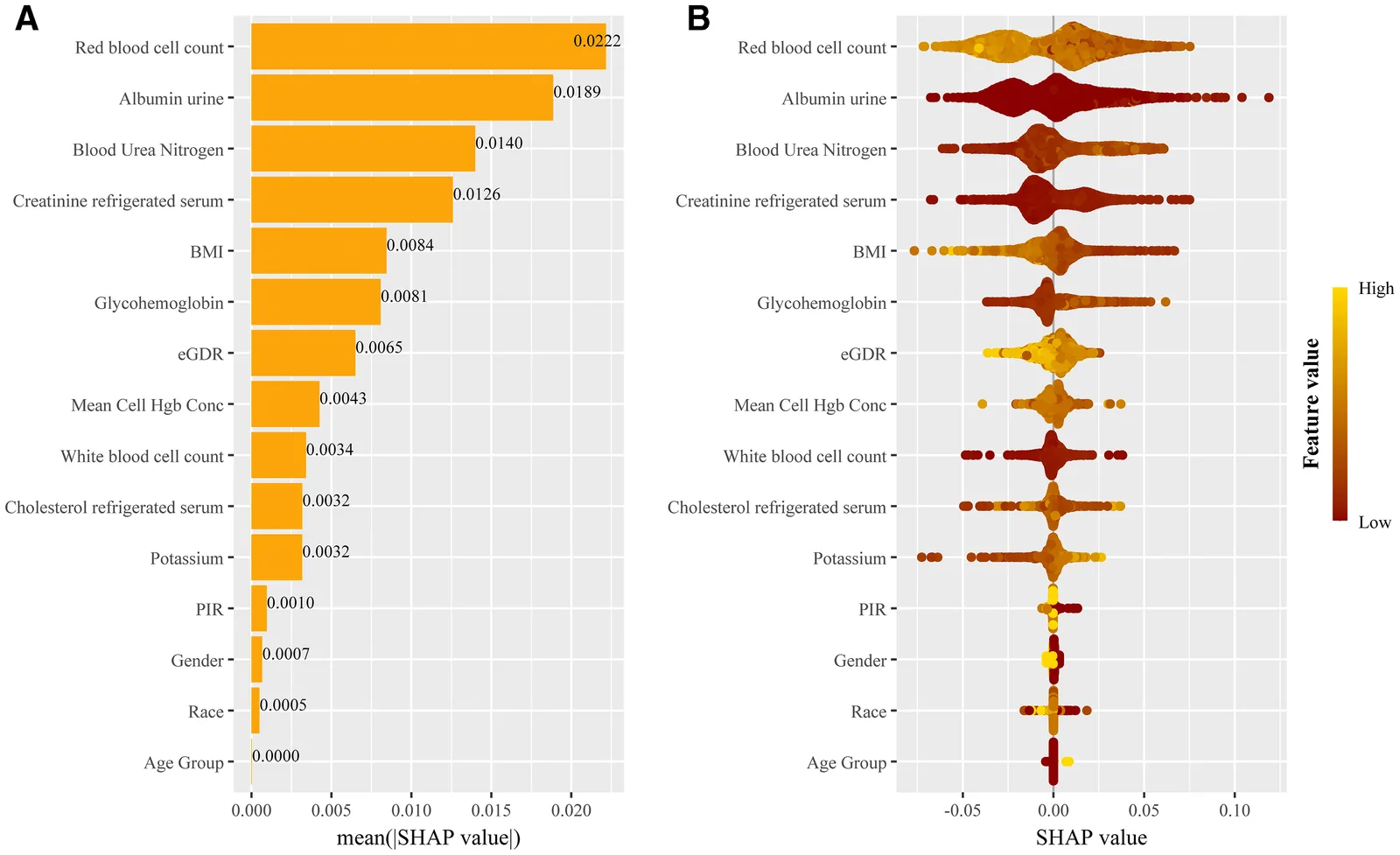
Explanation of machine learning models using SHAP summary and dependence plots. (A) Bar plot showing the mean absolute SHAP values and ranking the features by their predictive importance. (B) A SHAP summary plot showing the direction and strength of each feature’s association with the risk of undergoing cataract surgery. SHAP = SHapley Additive exPlanations.

### 3.4. Analysis of stratified patterns in cataract surgery

To evaluate the incidence of cataract surgery across different subgroups, we conducted an analysis (Fig. 4). The results revealed a dose-dependent inverse association between eGDR quartiles and the incidence of cataract surgery (Fig. 4A). The fourth quartile (Q4) showed the lowest surgery incidence rate (45.71%), whereas the second quartile (Q2) showed the highest (53.9%). Figure 4B shows the incidence of cataract surgery, which is stratified by age and PIR. This confirms an overall increase in the incidence of surgery related to age. Interestingly, the incidence of cataract surgery decreased progressively among individuals under 40 years of age across the low, medium, and high PIR groups. In contrast, among individuals aged 40 to 60, the incidence of cataract surgery increased and stabilized in the middle and high PIR categories. Among individuals over 60, the highest incidence was in the middle PIR group, and the lowest was in the high PIR group. These stratified patterns reveal the dynamic correlations between metabolic regulation (eGDR), socioeconomic status (PIR), and age-related cataract surgery.

**Figure 4.**
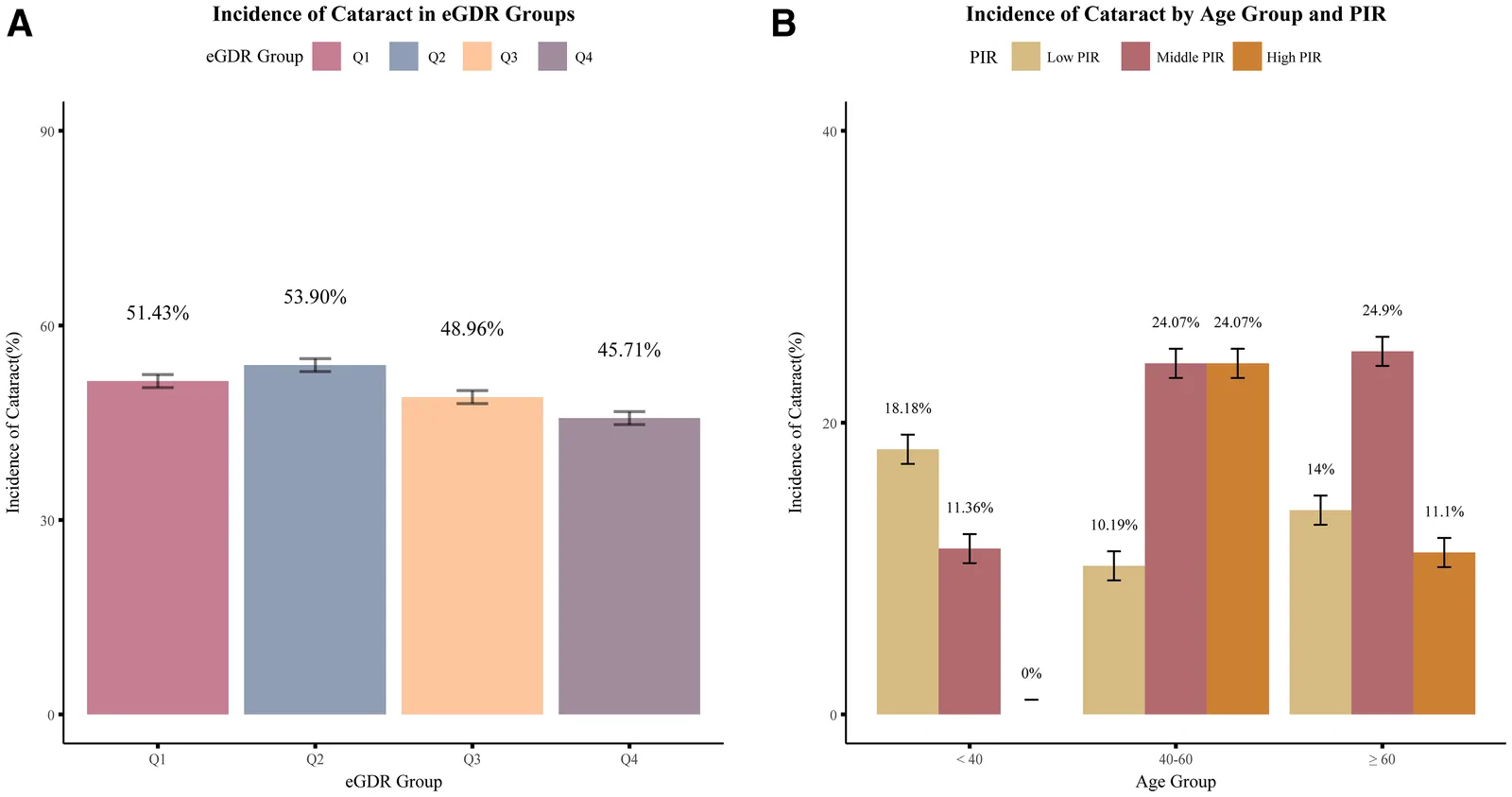
Incidence of cataract surgery across different subgroups. (A) Bar graph showing the incidence of cataract surgery across eGDR quartiles (Q1–Q4). Rates gradually decrease with increasing eGDR: 51.43% in Q1, 53.90% in Q2, 48.96% in Q3, and 45.71% in Q4. (B) Bar graph displaying cataract surgery incidence by age group and poverty-to-income ratio (PIR). The incidence increases with advancing age. The highest rates are observed in the middle PIR group for all age categories, particularly among participants aged ≥60 years (24.9%).

### 3.5. Relationship between eGDR and cataract surgery with subgroup stratification using restricted cubic splines (RCS)

The relationship between eGDR and cataract surgery was analyzed using RCS with complete covariate adjustment (Fig. 5). In the overall population, a significant inverse linear relationship was observed between eGDR and cataract surgery (*P* overall < .001; *P* nonlinear = .085). The RCS curve identified a critical threshold at eGDR = 5.693, above which each unit increase in eGDR provided incremental protection against the risk of cataract surgery (Fig. 5A).

**Figure 5.**
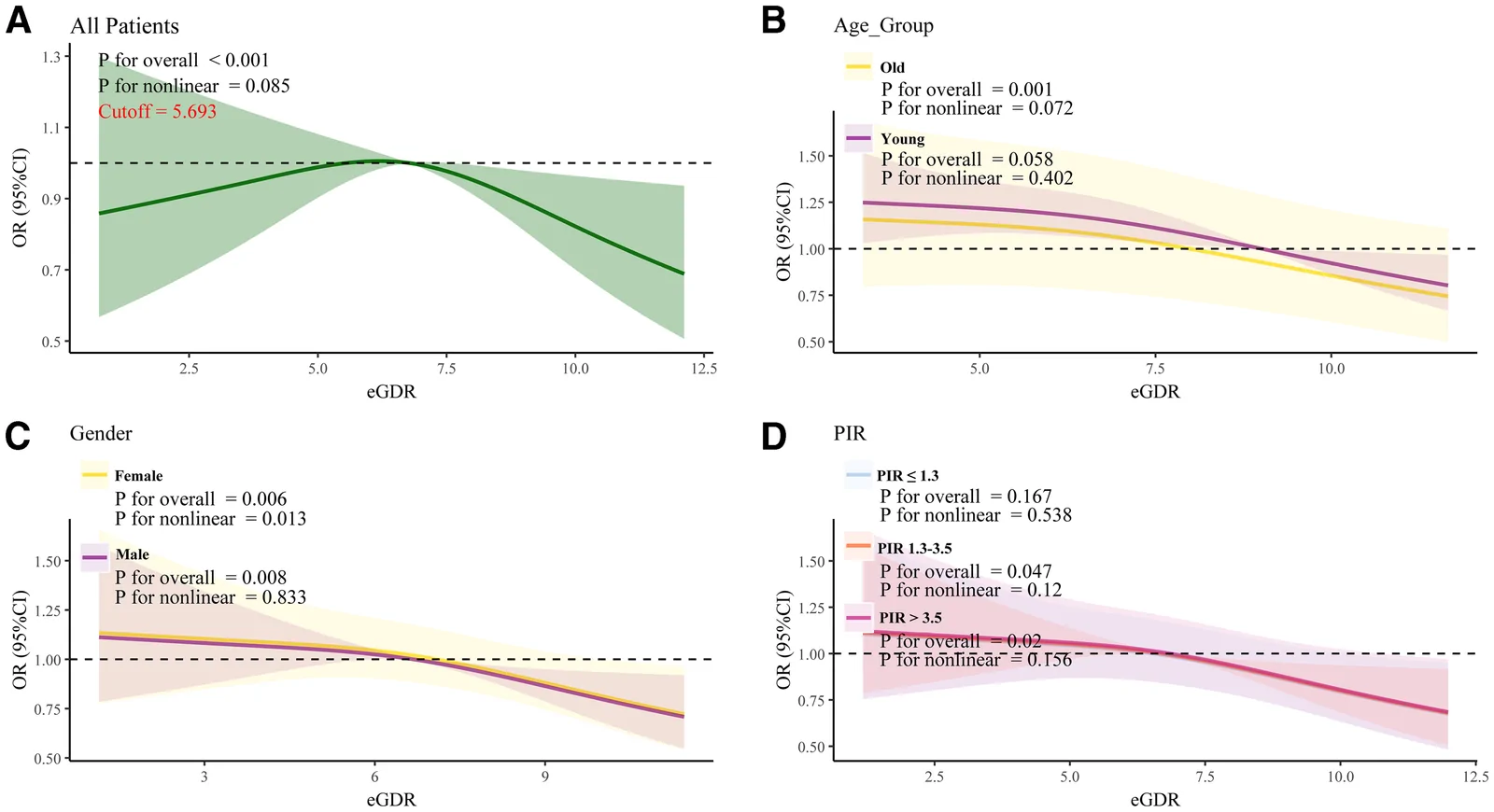
Restricted cubic spline (RCS) analysis of the association between eGDR and cataract surgery rate. (A) Overall association between eGDR and cataract surgery rate in all participants, showing a significant relationship (*P* < .001) with a nonlinear trend (*P* = .085) and an inflection point at an eGDR cutoff of 5.693. (B–D) Stratified RCS analyses by age group, gender, and PIR. Significant inverse associations between eGDR and cataract surgery risk were observed in the older age group, both genders, and the middle and high PIR subgroups. Covariates: age group, gender, race, family PIR, white blood cell count, BMI, blood urea nitrogen, urine albumin, total cholesterol, glycated hemoglobin, red blood cell count, MCHC, potassium, creatinine. BMI = body mass index, eGDR = estimated glucose disposal rate, MCHC = mean corpuscular hemoglobin concentration, PIR = poverty-to-income ratio.

Age-stratified analyses revealed a divergence: a robust linear inverse trend was observed in older adults (*P* overall = .001; *P* nonlinear = .072), in contrast to nonsignificant associations in younger populations (*P* overall = .058; *P* nonlinear = .402) (Fig. 5B). Gender-specific patterns emerged, with females showing nonlinear protection (*P* overall = .006; *P* nonlinear = .013) as opposed to linear associations in males (*P* overall = .008; *P* nonlinear = .833) (Fig. 5C). Socioeconomic gradients were apparent: low-income subgroups exhibited null associations (*P* overall = .167; *P* nonlinear = .538), while moderate-income groups demonstrated linear protection (*P* overall = .047; *P* nonlinear = .12), and high-income populations showed stronger inverse associations (*P* overall = .02; *P* nonlinear = .156) (Fig. 5D). Collectively, these subgroup analyses support eGDR as a potential protective factor against cataract surgery in the elderly.

### 3.6. Relationship between eGDR and cataract surgery across baseline subgroups

A multivariable logistic regression analysis was carried out to examine the correlation between eGDR and the history of cataract surgery among various subgroups based on baseline characteristics (Fig. 6). In the overall analysis, the highest eGDR quartile (Q4) showed a significantly lower incidence of cataract surgery compared to the lowest quartile (Q1) (OR = 0.8, 95% CI: 0.65–0.97, *P* = .025), confirming eGDR as a protective factor against cataract surgery. Subgroup analyses revealed different protective associations of the highest eGDR quartile (Q4 vs Q1): older adults (OR = 0.82, 95%CI:0.67–1.01, *P* = .065) and younger populations (OR = 0.46, 95%CI:0.17–1.2, *P* = .113) showed nonsignificant trends, while gender-specific reductions were observed in males (OR = 0.76, 95%CI:0.56–1.02, *P* = .067) but not in females (OR = 0.85, 95%CI:0.65–1.12, *P* = .25). Racial disparities emerged with significant protection among Mexican Americans (OR = 0.52, 95%CI:0.27–1, *P* = .049) and non-Hispanic Black individuals (OR = 0.44, 95%CI:0.23–0.86, *P* = .016), in contrast to null effects among non-Hispanic Whites (OR = 0.97, 95%CI:0.76–1.23, *P* = .793). PIR-stratified analyses showed nonsignificant trends in low- (OR = 0.76, 95%CI:0.52–1.11), middle- (OR = 0.82, 95%CI:0.61–1.09), and high PIR groups (OR = 0.8, 95%CI:0.53–1.23). Smoking status modulated the effects, with smokers showing a significant reduction in incidence (OR = 0.71, 95%CI:0.52–0.96, *P* = .024) compared to nonsmokers (OR = 0.88, 95%CI:0.68–1.16). Paradoxical patterns emerged in diabetes status: nondiabetics (OR = 0.96, 95%CI:0.76–1.22) and prediabetics (OR = 2.4, 95%CI:0.51–11.26) showed neutral associations. However, those without CVD showed protection (OR = 0.78, 95%CI:0.63–0.96, *P* = .021), which was absent in individuals with preexisting disease (OR = 1.09, 95%CI:0.56–2.1).

**Figure 6.**
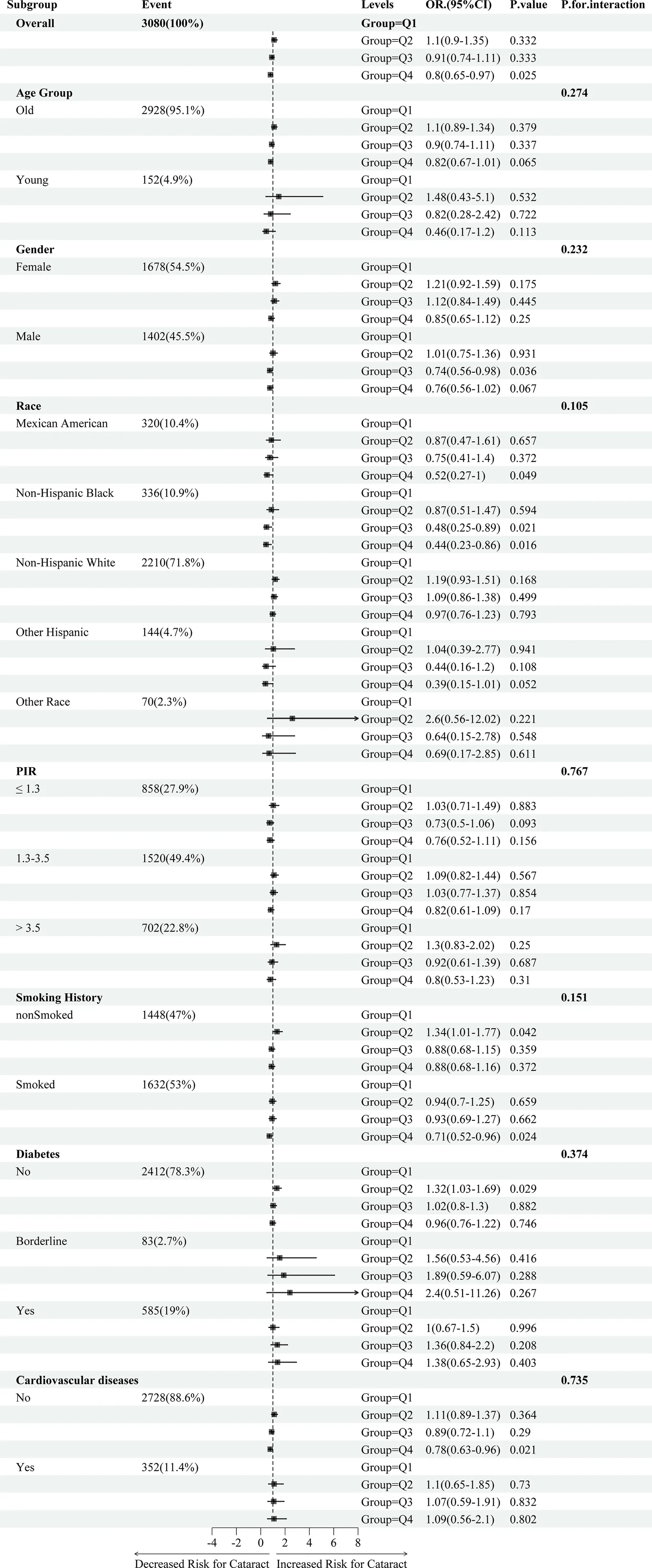
Subgroup analyses of the association between eGDR score and cataract surgery rate. eGDR Score was examined as a continuous variable. In terms of PIR, PIR ≤ 1.3: low PIR, 1.3 < PIR ≤ 3.5: middle PIR, PIR > 3.5: high PIR. Covariates: age group, gender, race, family PIR, white blood cell count, BMI, urea nitrogen, urine albumin, total cholesterol, glycated hemoglobin, red blood cell count, MCHC, potassium, creatinine. BMI = body mass index, eGDR = estimated glucose disposal rate, MCHC = mean corpuscular hemoglobin concentration, PIR = poverty-to-income ratio

### 3.7. Robustness validation through sensitivity analysis

To assess the reliability of the findings, sensitivity analyses were conducted by excluding individuals with diabetes, confirming the robustness of the conclusions. PSM analysis matched participants based on age groups, gender, race, and family PIR, resulting in a balanced cohort of 2356 participants (1178 cases and 1178 controls). The inverse association between eGDR and cataract surgery remained significant (*P* = .038; detailed in Table S4, Supplemental Digital Content 4). Multivariable linear regression models adjusted for age groups, gender, race, PIR, white blood cell count, BMI, blood urea nitrogen, urinary albumin, total cholesterol, HbA1c, red blood cell count, MCHC, potassium, and creatinine demonstrated persistent inverse associations. In unadjusted analyses, an increase of one unit in eGDR (treated as a continuous variable) was associated with a reduced rate of cataract surgery (OR = 0.966, 95% CI: 0.933–1.001, *P* = .0586). Consistent trends were observed in the adjusted models (Model 1: OR = 0.966, 95% CI: 0.932–1.002, *P* = .0606; Model 2: OR = 0.923, 95% CI: 0.88–0.969, *P* = .0012). Quartile-based analyses revealed dose-dependent protection: Q4 vs Q1 showed an OR of 0.815 (95%CI:0.649–1.025, *P* = .0805) in the crude model, an OR of 0.817 (95%CI:0.647–1.031, *P* = .0892) in Model 1, and an OR of 0.656 (95%CI:0.486–0.885, *P* = .0058) in Model 2. Significant linear trends were observed across all models (crude: *P* trend = .0358; Model 1: *P* trend = .0333; Model 2: *P* trend = .0022; shown in Table S5, Supplemental Digital Content 5). These results confirm the consistent protective association between eGDR and cataract surgery when using different analytical approaches. This reinforces the validity of the study.

## 4. Discussion

Cataracts rank among the top causes of visual impairment globally, significantly impacting the quality of life in affected individuals.^[1,3,4]^ This population-based study establishes the eGDR as an independent protective factor against the risk of cataract surgery, with a 4.7% reduction in the OR per unit increase in eGDR (OR = 0.953, 95% CI: 0.926–0.981). The robustness of this association persists across machine learning algorithms and traditional regression models, verifying the advantage of eGDR over separate metabolic parameters. Subgroup analyses reveal critical demographic interactions: the protective effect strengthens with age, shows gender differences (with a predominance among females), and shows socioeconomic stratification, particularly in low-income populations, consistent with the previous study.^[25]^ These findings are consistent with previous studies on the role of eGDR in systemic diseases.^[14–16,18]^ Interestingly, our study now extends its clinical relevance to ophthalmology, indicating its potential to play a crucial role in preventing illness.

To date, no specific studies have examined the relationship between eGDR and cataract surgery. However, diabetes-related hyperglycemia and IR (a characteristic feature of prediabetes) are well-known risk factors for cataract formation. A significant, strong correlation was found between aqueous humor glucose levels and blood glucose levels.^[26]^ Previous studies have emphasized the effect of IR on lens clouding and cataract development, indicating that metabolic imbalance might worsen the situation.^[27]^ This study identified an inverse association at the 5.693 mg/kg/minutes eGDR threshold, indicating a crucial turning point where IR substantially impacts the need for cataract surgery. From a biological standpoint, this cutoff plausibly reflects a threshold of insulin sensitivity below which chronic hyperinsulinemia, compensatory hyperglycemia, non-enzymatic glycation of lens proteins, and low-grade systemic inflammation become sufficient to accelerate lens opacification and cataract formation. In clinical practice or health interventions, maintaining or increasing the eGDR level of individuals above 5.693 may be more effective in preventing cataract surgery. This threshold closely matches the critical eGDR threshold identified in a previous study on cognitive decline.^[28]^ These results imply that eGDR could serve as a possible index for disease advancement across different physiological systems. This type of dynamic is well recorded in metabolic and endocrine disorders. Surpassing the acceptable threshold for IR could cause disrupted cerebral glucose metabolism,^[29]^ which might intersect with the pathogenesis of cataracts. IR can develop into diabetes,^[30]^ and elevated blood glucose levels in diabetics contribute to the development of cataracts.^[10,21,26]^ Identifying individuals at risk of reaching this metabolic threshold early allows for targeted interventions, such as lifestyle changes or pharmacological treatments, to reduce the need for cataract surgery,^[22,23,31]^ offering substantial clinical worth for preventive ophthalmology and public health approaches.

These findings have clinical implications that extend beyond academic interest. The inverse relationship between eGDR and cataract surgery suggests two practical approaches. First, eGDR has the potential to improve the early detection of high-risk individuals who are likely to benefit from enhanced lifestyle modification measures.^[25,28]^ Clinicians should take note of the particularly pronounced protective effect observed in women and older adults,^[25]^ two groups that are traditionally underrepresented in metabolic-ophthalmic research. Second, the socioeconomic gradient of the association between eGDR and cataract surgery underscores the need for targeted community programs in public health initiatives. Areas with high PIR may require interventions that address metabolic health and the social determinants of cataract surgery.^[25]^

There are three observations that present opportunities for translational work. Firstly, machine learning verification affirms the potential of eGDR for use in automatic risk prediction systems. Secondly, the eGDR composite index, which includes glycemic level, blood pressure, and waist-to-hip ratio, is adjustable, making it a beneficial characteristic for prevention strategies. This predictive effectiveness has also been noted in other diseases.^[11,13,16,17]^ Third, differences in eGDR levels and cataract surgery rates among different age groups suggest that metabolic vulnerability may follow different trajectories. Therefore, age-specific prevention strategies should be reevaluated. These results redefine eGDR. It serves not only as an indicator of IR but also as a system index with substantial ophthalmic applications. eGDR achieves this by merging metabolic, vascular, and anthropometric data into one easily interpretable index. This comprehensive risk evaluation may reduce the future need for age-related cataract surgery.

The current study has several strengths that enhance the reliability and significance of its results. Firstly, this study is the first to explore the relationship between eGDR levels and cataract surgery, offering new perspectives on the link between metabolism and cataracts. Secondly, the use of NHANES data, obtained from a strict multistage probability sampling design, guarantees that the findings are representative of the whole U.S. population. This method has been demonstrated to enhance the applicability and generalizability of the results and has been employed in many studies.^[32–35]^ Thirdly, controlling for potential confounders made it possible to more accurately assess the relationship between eGDR levels and cataract surgery. Therefore, the results have important implications for public health strategies focused on preventing cataract surgery.

However, several limitations must be acknowledged. First of all, the lack of wet lab experiments limits the ability to verify the mechanistic pathways via which eGDR impacts cataract formation. Being an observational study, our design precludes causal inference and may be prone to reverse causation. To confirm these results, future longitudinal research with larger and more varied populations is required. Moreover, the absence of clinical validation analysis raises concerns about the applicability of the results to real-world scenarios. Using multiple datasets may introduce batch effects, which could complicate the interpretation of the results and possibly confuse the observed relationships.

In summary, our findings indicate a significant negative correlation between eGDR and cataract surgery. This suggests that higher eGDR levels may protect against cataract surgery, especially in older adults and women. These findings highlight the significance of eGDR as a new IR indicator, which mirrors metabolic changes affecting cataract surgery. Future studies should focus on incorporating clinical validation and investigating the underlying biological mechanisms to further clarify the protective role of eGDR in cataract prevention. Such understandings could lead to focused interventions designed to cut down on cataract occurrence and enhance public health results.

## Author contributions

**Conceptualization:** Guoan Liu, Jinling Xie.

**Methodology:** Guoan Liu, Yanman Zhong, Jinling Xie.

**Resources:** Guoan Liu, Yanman Zhong.

**Visualization:** Guoan Liu, Jinling Xie.

**Data curation:** Yanman Zhong, Ruimei Qin, Jiaying Wang.

**Investigation:** Yanman Zhong.

**Software:** Ruimei Qin, Jiaying Wang.

**Formal analysis:** Jinling Xie.

**Project administration:** Jinling Xie.

**Supervision:** Jinling Xie.

**Validation:** Jinling Xie.

**Writing – original draft:** Guoan Liu, Yanman Zhong, Ruimei Qin.

**Writing – review & editing:** Jinling Xie.














