# Supplementary material for: Association between estimated glucose disposal rate and history of cataract surgery: A population-based cross-sectional study from NHANES
Source: Medicine (Baltimore). 2026 Oct 30;105(44):e51292. doi: 10.1097/MD.0000000000051292 (PMC13727194; doi:10.1097/MD.0000000000051292)
Supplement: Supplementary file 1 [file medi-105-e51292-s002.docx]

| **Table S1.** Multiple imputation before-after comparison of key variables. | | | | |
| --- | --- | --- | --- | --- |
|  | **BeforeMI** | **AfterMI** | **Overall** | **p-value** |
|  | **(N=13252)** | **(N=13252)** | **(N=26504)** |  |
| **Disease** |  |  |  |  |
| Cataract | 1565 (12 %) | 1565 (12 %) | 3130 (12 %) | 1 |
| Normal | 11687 (88 %) | 11687 (88 %) | 23374 (88 %) |  |
| **Age Years** |  |  |  |  |
| Mean (SD) | 55 (± 18) | 55 (± 18) | 55 (± 18) | 1 |
| **Age Group** |  |  |  |  |
| Old | 5940 (45 %) | 5940 (45 %) | 11880 (45 %) | 1 |
| Young | 7312 (55 %) | 7312 (55 %) | 14624 (55 %) |  |
| **Gender** |  |  |  |  |
| Female | 6787 (51 %) | 6787 (51 %) | 13574 (51 %) | 1 |
| Male | 6465 (49 %) | 6465 (49 %) | 12930 (49 %) |  |
| **Race** |  |  |  |  |
| Mexican American | 2455 (19 %) | 2455 (19 %) | 4910 (19 %) | 1 |
| Non-Hispanic Black | 2506 (19 %) | 2506 (19 %) | 5012 (19 %) |  |
| Non-Hispanic White | 6967 (53 %) | 6967 (53 %) | 13934 (53 %) |  |
| Other Hispanic | 821 (6 %) | 821 (6 %) | 1642 (6 %) |  |
| Other Race | 503 (4 %) | 503 (4 %) | 1006 (4 %) |  |
| **PIR** |  |  |  |  |
| <= 1.3 | 3414 (26 %) | 3661 (28 %) | 7075 (27 %) | 0.999 |
| 1.3-3.5 | 4841 (37 %) | 5200 (39 %) | 10041 (38 %) |  |
| > 3.5 | 4120 (31 %) | 4391 (33 %) | 8511 (32 %) |  |
| Missing | 877 (6.6%) | 0 (0%) | 877 (3.3%) |  |
| **Smoking History** |  |  |  |  |
| nonSmoked | 6663 (50 %) | 6663 (50 %) | 13326 (50 %) | 1 |
| Smoked | 6575 (50 %) | 6575 (50 %) | 13150 (50 %) |  |
| Missing | 14 (0.1%) | 14 (0.1%) | 28 (0.1%) |  |
| **BMI** |  |  |  |  |
| Mean (SD) | 29 (± 6.2) | 29 (± 6.2) | 29 (± 6.2) | 1 |
| Missing | 116 (0.9%) | 0 (0%) | 116 (0.4%) |  |
| **Weight** |  |  |  |  |
| Mean (SD) | 66 (± 4.1) | 66 (± 4.1) | 66 (± 4.1) | 0.78 |
| Missing | 313 (2.4%) | 0 (0%) | 313 (1.2%) |  |
| **Waist** |  |  |  |  |
| Mean (SD) | 99 (± 15) | 99 (± 15) | 99 (± 15) | 1 |
| **Diabetes** |  |  |  |  |
| No | 11298 (85 %) | 11307 (85 %) | 22605 (85 %) | 1 |
| Borderline | 242 (2 %) | 242 (2 %) | 484 (2 %) |  |
| Yes | 1702 (13 %) | 1703 (13 %) | 3405 (13 %) |  |
| Missing | 10 (0.1%) | 0 (0%) | 10 (0.0%) |  |
| **Hypertension** |  |  |  |  |
| No | 8130 (61 %) | 8130 (61 %) | 16260 (61 %) | 1 |
| Yes | 5122 (39 %) | 5122 (39 %) | 10244 (39 %) |  |
| **Cardiovascular diseases** |  |  |  |  |
| No | 12431 (94 %) | 12431 (94 %) | 24862 (94 %) | 1 |
| Yes | 740 (6 %) | 740 (6 %) | 1480 (6 %) |  |
| Missing | 81 (0.6%) | 81 (0.6%) | 162 (0.6%) |  |
| **Albumin urine** |  |  |  |  |
| Mean (SD) | 55 (± 460) | 58 (± 480) | 57 (± 470) | 0.994 |
| Missing | 116 (0.9%) | 0 (0%) | 116 (0.4%) |  |
| **Hemoglobin** |  |  |  |  |
| Mean (SD) | 14 (± 1.5) | 14 (± 1.5) | 14 (± 1.5) | 0.999 |
| Missing | 23 (0.2%) | 0 (0%) | 23 (0.1%) |  |
| **Red blood cell count** |  |  |  |  |
| Mean (SD) | 4.7 (± 0.51) | 4.7 (± 0.51) | 4.7 (± 0.51) | 0.999 |
| Missing | 23 (0.2%) | 0 (0%) | 23 (0.1%) |  |
| **Mean Cell Hgb Conc** |  |  |  |  |
| Mean (SD) | 34 (± 0.95) | 34 (± 0.95) | 34 (± 0.95) | 1 |
| Missing | 23 (0.2%) | 0 (0%) | 23 (0.1%) |  |
| **Mean cell hemoglobin** |  |  |  |  |
| Mean (SD) | 31 (± 2.2) | 31 (± 2.2) | 31 (± 2.2) | 1 |
| Missing | 23 (0.2%) | 0 (0%) | 23 (0.1%) |  |
| **White blood cell count** |  |  |  |  |
| Mean (SD) | 7.3 (± 2.6) | 7.3 (± 2.6) | 7.3 (± 2.6) | 1 |
| Missing | 23 (0.2%) | 0 (0%) | 23 (0.1%) |  |
| **Mean platelet volume** |  |  |  |  |
| Mean (SD) | 8.0 (± 0.89) | 8.0 (± 0.89) | 8.0 (± 0.89) | 1 |
| Missing | 23 (0.2%) | 0 (0%) | 23 (0.1%) |  |
| **Hematocrit** |  |  |  |  |
| Mean (SD) | 42 (± 4.4) | 42 (± 4.4) | 42 (± 4.4) | 0.999 |
| Missing | 23 (0.2%) | 0 (0%) | 23 (0.1%) |  |
| **C reactive protein** |  |  |  |  |
| Mean (SD) | 0.47 (± 0.90) | 0.47 (± 0.90) | 0.47 (± 0.90) | 1 |
| Missing | 6 (0.0%) | 0 (0%) | 6 (0.0%) |  |
| **Total Cholesterol** |  |  |  |  |
| Mean (SD) | 200 (± 42) | 200 (± 42) | 200 (± 42) | 1 |
| Missing | 7 (0.1%) | 0 (0%) | 7 (0.0%) |  |
| **Blood Urea Nitrogen** |  |  |  |  |
| Mean (SD) | 14 (± 6.4) | 14 (± 6.4) | 14 (± 6.4) | 1 |
| **Total Protein** |  |  |  |  |
| Mean (SD) | 7.2 (± 0.50) | 7.2 (± 0.50) | 7.2 (± 0.50) | 1 |
| **Total Bilirubin** |  |  |  |  |
| Mean (SD) | 0.75 (± 0.32) | 0.75 (± 0.35) | 0.75 (± 0.34) | 0.369 |
| Missing | 2004 (15.1%) | 0 (0%) | 2004 (7.6%) |  |
| **Chloride** |  |  |  |  |
| Mean (SD) | 100 (± 3.0) | 100 (± 3.0) | 100 (± 3.0) | 1 |
| **Aspartate aminotransferase AST** |  |  |  |  |
| Mean (SD) | 26 (± 13) | 26 (± 13) | 26 (± 13) | 1 |
| **Total Calcium** |  |  |  |  |
| Mean (SD) | 9.5 (± 0.38) | 9.5 (± 0.38) | 9.5 (± 0.38) | 1 |
| **Creatinine refrigerated serum** |  |  |  |  |
| Mean (SD) | 0.92 (± 0.41) | 0.93 (± 0.42) | 0.93 (± 0.41) | 0.00402 |
| Missing | 2004 (15.1%) | 0 (0%) | 2004 (7.6%) |  |
| **Alanine aminotransferase ALT** |  |  |  |  |
| Mean (SD) | 25 (± 18) | 25 (± 18) | 25 (± 18) | 1 |
| **Cholesterol refrigerated serum** |  |  |  |  |
| Mean (SD) | 200 (± 42) | 200 (± 42) | 200 (± 42) | 1 |
| **Lactate Dehydrogenase LDH** |  |  |  |  |
| Mean (SD) | 130 (± 28) | 130 (± 28) | 130 (± 28) | 0.398 |
| Missing | 2004 (15.1%) | 0 (0%) | 2004 (7.6%) |  |
| **Albumin refrigerated serum** |  |  |  |  |
| Mean (SD) | 4.2 (± 0.35) | 4.2 (± 0.35) | 4.2 (± 0.35) | 1 |
| **Potassium** |  |  |  |  |
| Mean (SD) | 4.0 (± 0.36) | 4.0 (± 0.36) | 4.0 (± 0.36) | 1 |
| **Sodium** |  |  |  |  |
| Mean (SD) | 140 (± 2.5) | 140 (± 2.5) | 140 (± 2.5) | 1 |
| **Triglycerides refrig serum** |  |  |  |  |
| Mean (SD) | 150 (± 110) | 150 (± 110) | 150 (± 110) | 1 |
| **Glycohemoglobin** |  |  |  |  |
| Mean (SD) | 5.7 (± 1.1) | 5.7 (± 1.1) | 5.7 (± 1.1) | 1 |
| **eGDR** |  |  |  |  |
| Mean (SD) | 7.8 (± 2.6) | 7.8 (± 2.6) | 7.8 (± 2.6) | 1 |
