# Supplementary material for: Association between estimated glucose disposal rate and history of cataract surgery: A population-based cross-sectional study from NHANES
Source: Medicine (Baltimore). 2026 Oct 30;105(44):e51292. doi: 10.1097/MD.0000000000051292 (PMC13727194; doi:10.1097/MD.0000000000051292)
Supplement: Supplementary file 2 [file medi-105-e51292-s005.docx]

| **Table S5.** Sensitivity analysis: logistic regression analysis of the association between eGDR exposure and cataract surgery. | | | | | | | | | | | |
| --- | --- | --- | --- | --- | --- | --- | --- | --- | --- | --- | --- |
| **Characterisitic** | **Exposure cutoff** |  |  | **Crude Model** |  |  | **Model1** |  |  | **Model2** |  |
|  |  | **Case (%)** | **OR** | **95%CI** | **P-value** | **OR** | **95%CI** | **P-value** | **OR** | **95%CI** | **P-value** |
| eGDR |  |  |  |  |  |  |  |  |  |  |  |
| eGDR continuous |  |  | 0.966 | (0.933,1.001) | 0.0586 | 0.966 | (0.932,1.002) | 0.0606 | 0.923 | (0.88,0.969) | 0.0012 |
| eGDR quantile |  |  |  |  |  |  |  |  |  |  |  |
| Q1(low) | < 5.61 | 589(25%) | Ref | Ref |  | Ref | Ref |  | Ref | Ref |  |
| Q2 | 5.61-< 7.36 | 589(25%) | 1.056 | (0.84,1.327) | 0.6408 | 1.052 | (0.834,1.327) | 0.6669 | 0.881 | (0.679,1.144) | 0.3435 |
| Q3 | 7.36-< 9.36 | 589(25%) | 0.897 | (0.714,1.127) | 0.3512 | 0.871 | (0.691,1.098) | 0.2434 | 0.754 | (0.583,0.975) | 0.0317 |
| Q4(high) | ≥ 9.36 | 589(25%) | 0.815 | (0.649,1.025) | 0.0805 | 0.817 | (0.647,1.031) | 0.0892 | 0.656 | (0.486,0.885) | 0.0058 |
| p for trend |  |  |  |  | 0.0358 |  |  | 0.0333 |  |  | 0.0022 |
| OR, odds ratio; CI, confidence intervals;, eGDR,Estimated Glucose Disposal Rate | | | | | | | | | | | |
| eGDR: Q1: eGDR < 5.61; Q2: 5.61 ≤ eGDR < 7.36; Q3: 7.36 ≤ eGDR < 9.36; Q4: eGDR ≥ 9.36 | | | | | | | | | | | |
| The Crude Model was non-adjusted  The Model 1 was adjusted by Age Group,Gender,Race,PIR The Model 2 was adjusted by Age Group,Gender,Race,PIR,White blood cell count,BMI,Blood Urea Nitrogen,Albumin urine,Cholesterol refrigerated serum, Glycohemoglobin, Red blood cell count,Mean Cell Hgb Conc, Potassium, Creatinine refrigerated serum | | | | | | | | | | | |
